# A comparison of career satisfaction amongst dental healthcare professionals across three health care systems: Comparison of data from the United Kingdom, New Zealand and Trinidad & Tobago

**DOI:** 10.1186/1472-6963-6-32

**Published:** 2006-03-14

**Authors:** Rahul Naidu, J Tim Newton, Katie Ayers

**Affiliations:** 1 School of Dentistry, Faculty of Medical Sciences, The University of the West Indies, Trinidad and Tobago, West Indies; 2 Oral Health Services Research & Dental Public Health, King's College London School of Dentistry, King's College London, UK; 3 Department of Oral Health, University of Otago, Dunedin, New Zealand

## Abstract

**Background:**

The aim of this study was to compare the expressed levels of career satisfaction of three groups of comparable dental healthcare professionals, working in Trinidad, the United Kingdom and New Zealand.

**Methods:**

Three questionnaire surveys were carried out of comparable dental healthcare professionals. Dental nurses in Trinidad and dental therapists in the UK and New Zealand. Questionnaires were sent to all registered dental nurses or dental therapists.

**Results:**

Career satisfaction was lowest amongst Dental Therapists working in Trinidad and Tobago. Approximately 59% of the Therapists working in New Zealand reported stated that they felt they were not a valued member of the dental team, the corresponding proportion in the United Kingdom was 32%, and for Trinidad 39%.

**Conclusion:**

Dental therapists working in different healthcare systems report different levels of satisfaction with their career.

## Background

The career development and career satisfaction expressed by dental professionals is an area which has attracted much recent research [1-5]. Most studies have focussed on hygienists and dental practitioners. In the United Kingdom (UK) Evans &Blinkhorn [6] found that the majority of dental hygienists (87%) found their work fulfilling and interesting, while a more recent national survey carried out by the British Dental Hygienists Association [7] also found high levels of job satisfaction among respondents. Similar findings are reported in surveys of UK hygienists qualifying from one particular school of dental hygiene [8,9] and amongst dental hygienists working outside the UK [10-14]. Amongst dental practitioners expressed levels of job and career satisfaction have generally been lower than those reported for hygienists. Low levels of career satisfaction have been reported amongst UKdentists [15-17] and dentists working in the United States [18,19].

The expressed levels of job satisfaction among dental therapists working in the UK was described by Gibbons et al [3] who reported that the average level was high. Further Newton and Gibbons [4] compared the satisfaction levels of three professional groups using a single item measure, revealing that dental practitioners had the lowest levels compared to dental hygienists and dental therapists. The present study compares the job satisfaction of three comparable groups of dental professionals working in the UK, Trinidad & Tobago and New Zealand using the same measure.

Trinidad & Tobago is a twin island state situated at the southern end of the Caribbean chain of islands. It is a democratic republic within the British Commonwealth having gained independence in 1962. The country's dental needs are currently served by dental professionals working in government health centres and in private practices. The Dental Council of Trinidad & Tobago presently only recognises two categories of dental professionals: dentists and dental nurses (the equivalent of dental therapists in the UK and New Zealand), with 216 and 50 registered and enrolled respectively [20]. The Dental Nurse Training Scheme in Trinidad & Tobago was modelled on the New Zealand program and began operating in 1976, producing its first graduates in 1978. The aim of the programme was to improve the dental operator to population ratio, improve access to dental services, particularly in the rural areas of the country and provide oral screenings and health promotion in schools [21]. Under the 1980 Dental Profession Act of Trinidad & Tobago, "A dental nurse is permitted to treat children (under 12 years old) only and such treatment shall be carried out in facilities or services operated or conducted by government or under direct or indirect supervision of a dentist in private clinics" [22]. These healthcare professionals have expressed confidence in their ability to perform their duties but low job satisfaction, due to poor working conditions and lack of opportunities for career development [20].

Currently, most dental therapists in New Zealand work within the School Dental Service treating children up to the age of 13 years. School dental services have provided free treatment since 1945 and it is estimated that more than 95% of New Zealand children are enrolled [23]. A recent review of the dental therapy workforce indicated several difficulties facing this profession: the lack of career structure, a narrow scope of practice, outdated facilities and inadequate remuneration. These difficulties are believed to be contributing factors to the poor recruitment and retention of dental therapists in New Zealand [24].

The provision of dental services in New Zealand is undergoing a period of change, largely due to the implementation of the Health Practitioners Competence Assurance (HPCA) Act in September 2004. This act allows for an expansion of the scope of practice of dental therapists, and also enables these oral health providers to move into private practice for the first time, hitherto dental therapists in New Zealand have been restricted to working in the publicly funded health sector, where remuneration is low. It is not yet known how these changes will affect the dental therapy in New Zealand though it is anticipated that many therapists will choose to shift their practice to the private sector where their potential earnings are higher.

Following the report of the Dental Auxiliary Review Group and the subsequent publication of a consultation document concerning professionals complementary to dentistry by the General Dental Council, recent legislation in the UK has allowed Dental Therapists to work in all sectors of oral healthcare. Prior to this dental therapists in the UK were limited to working in hospitals, community dental services or the armed forces where they were employed in salaried posts. This situation parallels that in New Zealand, and it is again anticipated that UK therapists will seek to enhance their income by working in the primary care sector where their earnings are likely to be paid on an item of service basis.

Three issues arise from a review of the reported career satisfaction of dental professions. First, there are few data available on the perceptions of some groups. Second, different studies have used different measures, making comparisons both within and across professions difficult. Third, there are no studies which have compared similar professional groups across countries (and by implication health care systems). The aim of the present study is to compare the expressed levels of career satisfaction of three groups of comparable dental healthcare professionals, working in Trinidad & Tobago, the UK and New Zealand.

## Methods

Three postal surveys were conducted. Parallel questionnaires, including a question about career satisfaction, were mailed to all dental therapists registered with the General Dental Council in the UK(n = 380), all dental nurses (n = 50) enrolled by the Trinidad & Tobago Dental Council and currently practising in Trinidad & Tobago, and all dental therapists on the Dental Council of New Zealand database (n = 716). Overall response rates for the surveys were: UK therapists 80%; Trinidad & Tobago nurses 76%; New Zealand 83%. Only dental therapists currently employed in that role were included in the analyses, reducing the sample size to: UK, 221 dental therapists; Trinidad & Tobago 38, New Zealand 502.

### Measure of satisfaction

Job satisfaction was determined by a single question. Participants were asked to rate their overall satisfaction with their career as a dental nurse or therapist (according to country) on a ten point scale with markers at each end where the value 1 was labelled "No satisfaction" and the value 10 labelled "Complete satisfaction".

In addition information was collected on the following

• Age of respondent

• Whether the respondent had ever taken a break in their career

• Whether the respondent felt a valued part of the dental team

### Analysis

Univariate analyses were conducted to compare dental personnel across the three countries, on the variables identified. Mean and median scores on the satisfaction scale were calculated and compared using the Kruskal-Wallis test (a non-parametric version of the oneway ANOVA) since the satisfaction data had a skewed distribution (the standard error of the skewness statistic was greater than twice the skewness). Age was treated as a continuous variable and compared using a one-way analysis of variance, since the distribution of the data was approximately normal. The proportion of individuals who had ever taken a career break was compared across countries, and the respondents perception of whether they felt a valued part of the dental team was treated as a dichotomous variable (all the time or most of the time vs some of the time, seldom or never) and compared using the Chi-square statistic.

In order to examine the relative importance of each variable in predicting satisfaction, a logistic regression analysis was conducted. The outcome variable was satisfaction score dichotomised around the median value for the sample (two categories were defined, scores of 1 to 7 inclusive, and scores of 8 to 10 inclusive. Age (as a continuous variable), whether the individual had taken a career break, place (entered as three separate dummy variables coding each of the three countries) and whether the individual felt a valued member of the dental team (dichotomised most and all of the time vs all other categories) were entered stepwise as predictor variables, in order of their simple correlation with the outcome variable. Additional variables were entered until there was no significant increase in the predictive utility of the variables. For each variable in the equation the following statistics were calculated: coefficient B, the standard error of B, significance, estimated odds ratio (exp [B]). For the final model the model chi-square and the log-likelihood statistic were calculated.

## Results

Table 1 summarises the univariate comparisons of dental professionals working in New Zealand, Trinidad & Tobago and in the UK. There was a significant difference in the age of the three groups of therapists. Post Hoc Scheffe tests revealed that each of the three means was significantly different from the other means. There were significant differences between the three groups of therapists in career satisfaction, post hoc Mann Whitney U tests comparing the three groups revealed that the Trinidad & Tobago based therapists had significantly lower career satisfaction than the other two groups. There was no significant difference in the career satisfaction of dental therapists in the UK and New Zealand.

The final logistic regression analysis model is summarised in Table 2. The Cox & Snell R-square for the final model was 0.106, and the log-likelihood was -945.8. Dental therapists working in New Zealand were almost twice as likely to express high levels of career satisfaction in comparison to the other two countries, while those working in Trinidad & Tobago were less likely than individuals from the other two countries to express high career satisfaction. Finally individuals from all countries who feel a valued member of the dental team express higher levels of career satisfaction.

## Discussion

The job satisfaction of three comparable groups of dental professionals working in different countries was compared, and found to be lowest in Trinidad & Tobago. These findings should be interpreted with some caution given the limitations of the study. This study adopted a single item measure of career satisfaction. Whilst this allows for comparison with other studies that have used similar single item measures for example [1,4], single item measures do not allow the investigation of the specifics of the work environment that are satisfying or dissatisfying. However a single item was chosen for its convenience in a postal survey and since it was felt that it provided a simple response format comparable across the three countries, and since it had been used in previous studies [1-3]. It is still possible however that the findings represent a cultural difference in the interpretation of 'career satisfaction. The three groups differed significantly in age, and the findings may in part be attributable to age, with younger participants being less satisfied with their career.

The extent to which an individual is satisfied with their job will be determined by many factors, including the pay and employment conditions. Country of work emerged as significant in the regression model, and is likely to act as a proxy measure for the working conditions, type of payment, healthcare system and experiences of the participants. However the present study did not investigate these explanatory variables in any depth. Surprisingly, whilst satisfaction was lowest amongst dental nurses in Trinidad & Tobago, this group had the highest proportion of members who felt a 'valued' member of the team. A feeling of being valued was lowest amongst dental therapists in New Zealand, suggesting that the satisfaction item was measuring something more than the perception of being valued in a team. The three countries in the present study place similar restrictions on the practice of dental therapy, and all three propose changes in the employment of this group of healthcare professionals but differ in the extent to which this change has been implemented. The UK system has taken the most steps towards changing the employment of dental therapists, followed by the New Zealand system. Career satisfaction was lowest amongst Dental Therapists in Trinidad and Tobago where the role is most restricted. Future research should address the extent to which the characteristics of the working environment impact upon job and career satisfaction. Research with dental practitioners has determined that system of remuneration, the characteristics of the working environment, and the type of service in which an individual works all exert an influence upon the practitioner's experience of their working life [15,16,25].

There is a need for further research addressing the impact of low career satisfaction on the dental workforce, including retention of workforce, the impact on the quality patient care and interactions with patients. It might be hypothesised that a dissatisfied workforce would be more likely to leave the career or be less motivated to deliver care of a high quality. Such associations have been found in studies of physicians [26] and rehabilitation professionals [27].

## Conclusion

Dental therapists working in different healthcare systems report different levels of satisfaction with their career, being lowest in Trinidad & Tobago. Career satisfaction in all three countries was related to feeling a valued member of the dental team.

## Competing interests

The author(s) declare that they have no competing interests.

## Authors' contributions

RN was responsible for the conduct of the survey in Trinidad & Tobago. JTN was responsible for the collection of data in the United Kingdom collated the data from the three countries and performed the statistical analysis. KA was responsible for the conduct of the survey in New Zealand. All authors contributed to the production of the manuscript.

## Pre-publication history

The pre-publication history for this paper can be accessed here:



## Figures and Tables

**Table 1:** Univariate comparisons of dental professionals working in Trinidad & Tobago, inNew Zealand and in the UK

	New Zealand (n = 502)	Trinidad & Tobago (n = 38)	United Kingdom (n = 221)	
*Age*^1^				
Mean (sd)	47.70^a ^(8.80)	38.76^b ^(6.09)	45.35^c ^(7.69)	F = 28.36 p < 0.001
*Career Break*				
Yes	412 (82%)	18 (47%) *	168 (76%)	Chi^2 ^= 26.9
No	89 (18%)	20 (53%) *	53 (24%)	p < 0.001
*Do you feel a valued member of staff?*				
All the time or most of the time	208 (41%) *	24 (63%)	151 (68%)	Chi^2 ^= 48.65
Some of the time, seldom or never	299 (59%) *	14 (37%)	70 (32%)	p < 0.001
*Career Satisfaction*^2 ^				
Mean (SD)	7.13 (1.95)^d^	5.21 (2.35)^e^	7.34 (1.94)^d^	Kruskall-Wallis Chi^2 ^= 30.5
Median	8	5	8	p < 0.001

1 Means with different superscripts are significantly different (post-hoc Scheffe test)

2 Means with different superscripts are significantly different (post-hoc Mann Whitney U tests)

* For Chi-square analysis, these cells have the largest corrected residuals suggesting that these cells make the largest contribution to the Chi-square value.

**Table 2:** Results of logistic regression analysis predicting career satisfaction (dichotomised around the median value (categories 1 to 7 inclusive versus 8 to 10 inclusive) as the dependent variable

Variables included in the analysis	Beta coefficient	Standard error	*P*	Exp(B)	95% CI of Exp(B)
*Country = New Zealand *(Comparison=all other participants)	0.67	0.21	<0.001	2.60	1.92 – 3.52
*Country=Trinidad & Tobago *(Comparison=all other participants)	-1.95	0.47	<0.001	0.14	0.06 – 0.36
*Feeling a Valued member of dental team* (Most or all the time vs all other participants)	1.49	0.20	<0.001	4.45	3.00 – 6.59

Log-likelihood -945.8; model Chi-square = 83.48 (*p *< 0.001)
